# Association between Haem and Non-Haem Iron Intake and Serum Ferritin in Healthy Young Women

**DOI:** 10.3390/nu10010081

**Published:** 2018-01-12

**Authors:** Isabel Young, Helen M. Parker, Anna Rangan, Tania Prvan, Rebecca L. Cook, Cheyne E. Donges, Kate S. Steinbeck, Nicholas J. O’Dwyer, Hoi Lun Cheng, Janet L. Franklin, Helen T. O’Connor

**Affiliations:** 1 Nutrition and Dietetics Group, School of Life and Environmental Sciences, Faculty of Science, The University of Sydney, Sydney, NSW 2006, Australia; iyou3908@uni.sydney.edu.au (I.Y.); anna.rangan@sydney.edu.au (A.R.); 2 Faculty of Health Science, Discipline of Exercise and Sports Science, The University of Sydney, Lidcombe, NSW 2414, Australia; rebecca.cook@sydney.edu.au (R.L.C.); nicholas.odwyer@sydney.edu.au (N.J.O.); helen.oconnor@sydney.edu.au (H.T.O.); 3 Charles Perkins Centre, The University of Sydney, Camperdown, NSW 2006, Australia; helen.cheng@health.nsw.edu.au; 4 Department of Statistics, Faculty of Science and Engineering, Macquarie University, Sydney, NSW 2113, Australia; tania.prvan@mq.edu.au; 5 School of Exercise Science, Sport and Health, Charles Sturt University, Bathurst, NSW 2795, Australia; cheyne_donges@outlook.com; 6 Academic Department of Adolescent Medicine, The Children’s Hospital at Westmead, Westmead, NSW 2145, Australia; kate.steinbeck@health.nsw.gov.au; 7 Sydney Medical School, Discipline of Child and Adolescent Health, The University of Sydney, Westmead, NSW 2145, Australia; janet.franklin@sswahs.nsw.gov.au; 8 Metabolism and Obesity Services, Royal Prince Alfred Hospital, Camperdown, NSW 2050, Australia

**Keywords:** haem iron, non-haem iron, iron deficiency, young women, serum ferritin

## Abstract

Iron is an essential micronutrient for human health and inadequate intake may result in iron deficiency (ID) or iron deficiency anaemia (IDA). Unlike other recent studies investigating iron status in young women, this cross-sectional study analysed dietary intake and biochemical data from healthy young (18–35 years) women (*n* = 299) to determine the association between both haem iron (HI) and non-haem iron (NHI) intakes and serum ferritin (SF). Dietary restraint and possible inflammation secondary to obesity were also measured and accounted for, and energy intake was adjusted for using the residuals method. Independent samples *t*-tests and chi-squared tests were performed, and factors found to be significantly different between iron replete (IR) and ID/IDA participants were analysed using general linear modelling. ID/IDA participants consumed significantly lower total energy than iron replete (IR) (*p* = 0.003). Lower energy intake was also associated with higher levels of dietary restraint (*p* = 0.001). Both HI and NHI were positively associated with SF with HI was found to be a stronger predictor (*β* = 0.128, *p* = 0.009) than NHI (*β* = 0.037, *p* = 0.028). The study demonstrates that intake of both HI and NHI, as well as adequate dietary energy, are associated with normal iron status levels in young women, and that restrained eaters may be at greater risk of low iron status.

## 1. Introduction

Iron is an essential micronutrient for human health, and is involved in DNA and enzyme synthesis, oxygen transportation, erythropoiesis, metabolism, and immune function [1,2]. Low iron status may result in iron deficiency anaemia (IDA) and is associated with poor physical, cognitive, and immune development and function [3]. Additionally, iron deficiency (ID) without IDA has also been associated with negative effects in adults, including lethargy, difficulty concentrating, and poor immune function [1,4].

Dietary iron is present in two forms, haem iron (HI) and non-haem iron (NHI), with HI being absorbed from the gut with greater efficiency [1,2,5]. The greater efficiency of absorption is due to specific haem transporters which enable HI to pass directly across cell membranes and into the bloodstream [2,5,6], whereas NHI is unable to utilise these transporters, requiring reduction of ferric iron to ferrous iron to occur prior to absorption [2]. Animal products, such as meat, poultry, and fish, are major dietary contributors of HI, comprising approximately 55–70% of the total iron content of these products [7,8]. The remainder of the iron in these food items, and iron present in non-animal products, such as legumes, breads, and cereals, including the elemental iron present in supplements and for food fortification, is NHI [1,5,6]. As a result, although HI typically makes up 10–15% of dietary iron intake in an omnivorous diet, it contributes 40% or more to the total iron absorbed by the body due to its higher absorptive capacity [9]. As such, the type of dietary iron (HI or NHI) may be a more important determinant of iron status than total dietary iron intake [10].

Young women of child bearing age (<50 years) are one of the most at-risk group for ID with a prevalence in Australia estimated to be 20% [11]. Young women are also at increased risk of IDA with 6.4% of Australian women compared to only 2.5% of young men reported to have IDA [12]. Women’s higher risk of ID can be attributed to menstrual losses, which can contribute significantly to ongoing iron depletion during the reproductive years [13]. Women also often have lower overall dietary intake and, in turn, iron intake when compared to men [14,15]. This is particularly important during pregnancy as during the third trimester iron requirements increase substantially to support the growth of the foetus [16]. Young women, in particular, are at risk of low iron intake due to the high proportion engaging in dieting behaviours, such as energy or food group restriction, and disordered eating [17] which may increase an individual’s risk of nutrient deficiencies [18,19]. Dietary restraint often involves reduced intake of red meat, and should be explored for when studying the relationship between diet and risk of ID/IDA.

Other factors may affect iron status in young women. The oral contraceptive pill, commonly used in this age group, protects against ID through reduced menstrual losses [20,21]. Inflammation secondary to a range of conditions, including obesity, is known to alter iron metabolism and is often associated with ID [22]. Inflammation can complicate diagnosis of iron status as serum ferritin (SF) (a key indicator of ID) is an acute phase reactant and may be falsely elevated in the presence of inflammation, potentially leading to missed diagnosis of ID [23]. As young women are one of the groups within the population who are at an increased risk of obesity [24], it is of clinical importance that obesity be accounted for when examining iron status studies.

Few studies have assessed intakes of both HI and NHI in conjunction with dieting habits and weight status. A recent study examined the contribution of HI intake to iron status in a large cohort of Australian women [25], however, in that study iron status was self-reported rather than measured haematologically. Furthermore, we sought to understand how HI and NHI intakes independently contributed towards iron status in young women. Therefore, this study aimed to explore the associations between intakes of both HI and NHI and the iron status of young women while taking into account dietary restraint, inflammation, and other confounders.

## 2. Materials and Methods

This is a secondary analysis of data collected in the Food, Mood and Mind Study [26]. This cross-sectional study recruited healthy young (18–35 years) women from urban (*n* = 200) and rural (*n* = 100) areas of New South Wales, Australia, of both normal weight (NW) (*n* = 150; body mass index (BMI): 18.5–24.9 kg/m^2^) and obese weight (OB) (*n* = 150; BMI: ≥30 kg/m^2^). The study was approved by Human Research Ethics Committees linked to local health district services and participating universities (protocol numbers: X10-0259, HREC/10/RPAH/455, and 2014/050). Written informed consent was obtained from all participants prior to participating in the study.

### 2.1. Inclusion and Exclusion Criteria

Women were eligible if they were healthy, with a BMI in either the normal weight or obese weight category, as per the World Health Organisation (WHO) guidelines (NW: BMI 18.5–24.9 kg/m^2^, OB: BMI ≥ 30 kg/m^2^) [27]. Volunteers were excluded if they reported any significant medical conditions, including metabolic, cardiac, renal or malignancy diseases, as well as disorders which cause gastrointestinal bleeding. Women who were pregnant or breastfeeding, regular blood donors (three or more donations per year), had donated blood within the previous three months, reported taking iron supplements, or those with known iron deficiency were also excluded. All potential participants were initially screened using a standardised pro forma via telephone.

### 2.2. Data Collection

Data collection included anthropometric measurements, a validated Food frequency questionnaire (FFQ) [28] and a fasting blood sample.

#### 2.2.1. Anthropometry

Anthropometric data were collected from participants in light clothing with no shoes. Weight was recorded on an electronic digital platform scale (PW-200KGL, Thebarton, Adelaide, Australia) to the nearest 0.1 kg. Height was recorded to the nearest 0.1 cm in duplicate with a stadiometer (213 portable stadiometer; SECA, Hamburg, Germany). Waist circumference was recorded to the nearest 0.1 cm in duplicate with a retractable metal tape (Lufkin W606PM; Cooper Industries, Sparks, NV, USA) according to International Diabetes Federation Guidelines [29]. All instruments used for measurement were calibrated and methods validated internally (height and weight) and externally (waist circumference).

#### 2.2.2. Blood Collection

A fasting (12 h) blood draw was conducted and biochemical markers of iron status (haemoglobin (HB) and SF) and inflammation (C-reactive protein (CRP) and alpha-1 acid glycoprotein (α1GP)) were measured by a National Association of Testing Authorities Australia (NATA) accredited diagnostic laboratory (https://www.nata.com.au/nata/, Sydney, Australia). SF was measured via automated immunoassay on COBAS 8000 e602 (Roche Diagnostics, Indianapolis, IN, USA). Absorption spectrophotometry and flow cytometry on ABBOTT CELL-DYN Sapphire (Abbott, Sydney, Australia) was used to measure Hb. CRP was measured using rate nephelometry on COBAS 8000 e602 and α1GP using immunoturbidimetry on Konelab 20XT Clinical Chemistry Analyser (Thermo Electron Oy, Vantaa, Finland) (using reagent from Thermo Fisher, Boston, MA, USA), with CRP > 5.0 mg/L and/or α1GP > 1.0 g/L indicative of raised levels of inflammation [23,30].

Participants were classified as iron deficient (ID) if ferritin was < 20 µg/L (reference range: 20–300), iron deficient anaemia (IDA) if haemoglobin was < 120 g/L (reference range: 120–150), or iron replete (IR) if ferritin > 20 µg/L and Hb > 120 g/L [31].

It is known that inflammation can elevate SF levels which may result in overestimation of iron stores and potential misclassification of participants as iron replete [23]. Therefore, raw SF were corrected as per the method from Northrop-Clewes et al. [23]. Correction factors applied were 0.77 when CRP only was raised, 0.53 when both CRP and α1GP were raised, and 0.75 when α1GP only was raised. Corrected SF levels were used to re-classify participants against the standard SF reference range.

#### 2.2.3. Food Frequency Questionnaire

A semi-quantitative FFQ, the Dietary Questionnaire for Epidemiological Studies Version 2, was used to assess the usual dietary intake [32]. This FFQ has been validated in Australia for measurement of dietary iron intake, and use in ethnically diverse populations [28,33]. The questionnaire includes 74 food items to determine usual portion sizes and types of common foods and beverages consumed including alcoholic beverages. FFQ analysis was automated and performed by the Victorian Cancer Council. Results were given as average daily intake of each food/beverage item (grams) and daily total energy and nutrient intakes.

The iron concentration (mg/100 g) of food items was not readily available from the FFQ results and, therefore, had to be calculated mathematically using simultaneous equations in MATLAB (The MathWorks, Inc., Natick, MA, USA) [34] with constraint, iron concentration ≥ 0. The iron content of each food item was rounded to two decimal places, and multiplied by the amount of each food (g/day) the participants consumed based on FFQ results. The sum of all iron intakes was then calculated to obtain a measurement for the total daily iron intake for each participant. This estimated total iron intake was compared to total iron intake per day from the automated FFQ results via a Bland-Altman plot [35] where the mean difference was found to be −0.004 mg/day with no obvious bias.

A total of 15 HI containing foods were identified in the FFQ including 12 meat products and three ‘mixed’ products (i.e., containing some meat). HI content of these foods was determined by using percentages published by Rangan et al., 1997 which are specific to the Australian food supply [8]. The values of HI (mg/g) of the wet weight of cooked food were originally analysed using the modified Hornsey method [36]. Percentages were averaged and applied to the iron content of each of the 12 meat products. The HI content of mixed products was calculated by determining the percentage of the food item that comprised meat and multiplying this by the HI content for the constituent meat type. The remaining iron in the HI foods was assumed to be NHI. Other iron-containing food items from a plant or other non-meat source were assumed to contain 100% NHI [2].

Food group intake was determined using the standard serve sizes from the Australian Guide to Healthy Eating (AGHE) [37]. Individual food items were grouped and their weights divided by their standard serve size. The number of serves for each food group was then added for each participant to provide total serves per day. Alcohol was excluded from discretionary items and included in its own food group as reported in the FFQ. Nutrient and food group data were adjusted for total energy intake using the residuals model [38].

#### 2.2.4. Other Information Collected

Participant characteristics, such as ethnicity, years of education, level of completed education, use of the oral contraceptive pill (OCP), and location (urban/rural) were collected. The International Physical Activity Questionnaire (IPAQ)—Short Form, was used to estimate physical activity using Metabolic Equivalent of Task (MET)-minutes per week [39]. Participants also completed the Three Factor Eating Questionnaire (TFEQ) to measure dietary restraint [40].

Basal metabolic rate (BMR) was calculated for each participant [2] using Schofield’s equation and used with reported energy intake to obtain a BMR: energy intake (EI) ratio. Low energy reporters were identified using the Goldberg equation using a cut off of 1.1 for habitual intake [41]. Participants were categorised into low energy reporters and plausible energy reporters.

### 2.3. Statistical Analysis

Prior to analysis of results, data were screened and participants with incomplete or extremely implausible datasets in the following areas were excluded: dietary intake and nutrient data (*n* = 10), biochemical data (*n* = 4), anthropometric data (*n* = 3), and extreme energy reporters (<3280 kJ, >19,500 kJ) (*n* = 12) [42,43,44]. The remaining data were used for statistical analysis (*n* = 270).

Analyses were undertaken using IBM SPSS Statistics for Windows, version 23 (IBM Corp., Armonk, NY, USA). Independent samples *t*-tests (Welch’s test when the homogeneity of variances assumption was violated) or independent samples median tests (when the normality assumption was violated) and chi-squared tests for homogeneity (or Fisher’s Exact Test) were used to compare continuous and categorical participant characteristics, respectively, between IR and ID/IDA participants. A logistic regression was performed to analyse the effect of education iron status and a one-way ANOVA was performed to analyse the association between energy intake and dietary restraint. General linear modelling (GLM) was undertaken to assess the association between HI and NHI (adjusted for total energy intake [38]) and SF adjusting for potential confounders (age, BMI, education, energy intake, and energy reporting status).

## 3. Results

This cross-sectional study recruited 299 healthy young (18–35 years) women. The prevalence of ID and IDA within the group was 26.3% (*n* = 71) and 5.9% (*n* = 16), respectively, with 67.5% of the group being IR (*n* = 183). Participants with ID and IDA were pooled (*n* = 87) and compared to IR, and their characteristics summarised in Table 1.

Participants were of varying education levels, with 56.3% having completed a tertiary degree (*n* = 152). The mean weight of participants was 77.9 ± 23.8 kg with a mean BMI of 28.5 ± 8.6 kg/m^2^. Age, ethnicity, location (urban/rural), total activity (METmin/week), anthropometric measurements, and dietary restraint were not significantly different between the IR and ID/IDA groups.

The distribution of participants across education groups was significantly different (*p* = 0.014). It was found that participants with secondary education as their highest qualification were twice as likely (odds ratio 2.1) to be ID/IDA than those with higher education (*χ*^2^(2) = 8.365, *p* = 0.015).

Participants with high dietary restraint had significantly lower reported energy intakes than those with low or moderate restraint. Mean energy intake for high restrainers was 6622 kJ, compared to 7556 kJ and 8148 kJ for moderate and low restrainers, respectively (*p* = 0.001). Dietary restraint was compared between OB and NW participants, which showed no significant weight-related differences.

There were significantly more low-energy reporters in the ID/IDA group (*p* = 0.005), with 70.1% compared to 51.9% for IR. Low-energy reporting was also significantly higher in the OB versus the NW groups (*χ*^2^(1) = 18.046, *p* < 0.0005), with 71.4% of OB participants reporting low energy intakes compared with 45.8% of NW participants (data not shown). There were also significantly more low energy reporters in the high dietary restraint group when compared to the low and moderate restraint groups (*p* = 0.029, *n* = 49(31.4%)).

The oral contraceptive pill was used by 32.3% of the cohort and was not found to be significantly different between the two iron status groups (*p* = 0.863). Although there were missing data for oral contraceptive pill use (*n* = 35), the proportion of missing data from both groups was not significant.

IR participants had higher average reported energy intakes than ID/IDA participants (Table 2). When comparing food and nutrient intakes that were unadjusted for energy, intakes of all nutrients (with the exception of calcium and vitamin C), meat, red meat, and grains and cereals, were significantly higher for IR participants (Table A1). When comparing unadjusted food groups intakes of meat, red meat and alcohol were significantly higher in IR participants than those with ID/IDA (Table A1).

After adjusting for energy (Table 2), IR participants were found to consume higher amounts of HI than ID/IDA participants, with mean intakes of 2.1 ± 1.0 mg and 1.8 ± 0.8 mg per day respectively (*p* = 0.043). Additionally, intakes of iron (*p* = 0.012) and zinc (*p* = 0.008) were significantly higher in the IR group compared to the ID/IDA group. Overall, when looking at food group data, there was a trend for higher consumption of meat in IR compared to ID/IDA participants (*p* = 0.058).

Intakes of HI and NHI, and higher levels of education were positively associated with SF (Table 3). HI had a stronger association with SF (*β* = 0.128) than NHI (*β* = 0.037). The overall model explained 5.8% of the variance in SF. The addition of energy intake into the model did not substantially affect the regression coefficients for HI and NHI intake.

## 4. Discussion

The main findings of this study were the significant, positive association between energy-adjusted HI and NHI intake and SF and total energy intake and SF on iron status in healthy young women. Energy-adjusted HI had a stronger association than NHI, indicating that iron bioavailability is an important consideration for maintaining normal iron status in young women during reproductive years. The negative effect of restricting energy intake on iron status also reinforces the importance of adequate energy consumption to healthy iron status. The proportion of women with ID and IDA within the sample was 25.6% and 5.6%, respectively, or approximately one third of participants having a sub-optimal iron status. This high prevalence of sub-optimal iron status, when compared to current estimates of 20% within the Australian population [11], confirms the relevance of examining this nutrient deficiency in young women. No associations were found between SF and BMI, OCP use, or level of physical activity; however, women with the lowest level of education (high school completion only) had lower SF than those with the highest level of education (tertiary degree completion). The overall model including energy adjusted HI and NHI and level of education only explained 5.8% of the variance in SF. Therefore, other factors, such as iron loss via menstruation and the presence of absorption enhancers and inhibitors for NHI [44,45], would be expected to contribute to the variance in SF levels.

HI was found to be an independent predictor of SF in healthy young women. This indicates that intake of HI containing products such as meat (beef, veal, lamb, poultry, and fish) makes a useful contribution to maintaining adequate SF levels. This is supported by the analysis of food groups, which showed a non-significant trend for greater energy-adjusted meat consumption (*p* = 0.058) in IR participants. While this association has been suggested previously [10,25], few studies have combined the use of biochemical markers to diagnose iron deficiency, or specifically identify both HI and NHI intakes. Although NHI was a significant predictor of SF in this study, it was more weakly associated with SF (*β* = 0.037) when compared to HI (*β* = 0.128). This is likely due to its lower absorptive capacity when compared to HI [5,9]. Despite this, the contribution of NHI is important to consider, especially for vegetarians and vegans who rely solely on NHI. In this case, the bioavailability of NHI can be increased by the concurrent intake of enhancers, such as ascorbic acid [45] or, conversely, inhibited by intake of phytates found in fibrous foods which bind to NHI in the gut, limiting its absorption [46]. The concurrent intake of both copper and zinc in specific mole ratios with iron can also cause decreases in iron absorption as the metals compete for transport across the gut [47]. The dual significance of both HI and NHI has also been reported in the French population [48], whilst the significance of NHI in the presence of absorption enhancers has also been noted in women from Central Mexico [49] and China [50].

Although biochemical evidence of zinc status was not reported in this study, zinc intake, although well above the RDI for women of this age group (8 mg/day), was found to be significantly different between the ID/IDA and IR groups, with IR participants consuming a significantly higher amount. This is consistent with previous research reporting that zinc intake increases with iron intake [51] and is likely due to both zinc and iron being found in similar foods, such as meat products and some fortified foods [52].

Another important finding from this study was the significant difference in reported energy intake between ID/IDA and IR groups. This is supported by other research which has found that increased total energy intake was associated with increased total iron intake [53]. The additional energy consumed by the IR group was not from any one particular food group, and any differences in energy-adjusted food group intake did not reach statistical significance. However, the main differences in consumption appeared to be in the meat group, especially red meat, with a non-significantly greater number of servings in the IR group, supporting the stronger association that HI had with SF (*p* = 0.058).

High dietary restraint was found to be associated with lower energy intakes, suggesting that this is a risk factor for maintaining iron status in young women. Numerous studies have reported on the link between dieting and risk for micronutrient deficiency and also the tendency for iron containing foods such as meats, especially red meats, being more heavily restricted than other food groups when dieting occurs in young women [17]. This is further supported by Cheng et al. (2013) who found that overweight and obese participants on energy restricted diets were unable to meet the recommended intake of iron, despite meal plan manipulation and dietary modelling [54]. Interestingly, no significant difference was found between OB and NW weight groups for dietary restraint. This suggests that high restraint resulting in decreased energy intake could have negative impacts on iron status regardless of body weight or BMI.

Tertiary education was also significantly associated with higher SF which may be a result of participants being more aware of their health, including the risk of iron deficiency during reproductive years, leading individuals to follow better quality diets [55]. It has been shown that socioeconomic status (SES) also including income and occupation have an impact on diet quality and iron status [55,56].

Strengths of this study included the use of biochemical markers to adjust for inflammation and for the diagnosis of iron status. The collection of biochemical data is important for ID/IDA diagnosis as women may not always be aware of their iron status [57], and this is a limitation for studies where iron status is self-reported [25]. Dietary restraint was also measured and accounted for, and participants were otherwise healthy, reducing confounding from medical conditions which compromise iron status. The FFQ used in the current study was specifically validated for use within the study population and for measuring iron intake [28,33]. HI and NHI content was calculated for each individual food item which allowed for both forms of iron to be analysed concurrently, enabling comparisons to be made about their respective and combined associations, analysis which has been identified as important in the future [25].

A limitation of the study was the use of an FFQ which is prone to both over and under-reporting of total energy intake [58,59]. The FFQ does not include information about the timing of food consumption and combinations of foods. This would have been useful to analyse the possible inhibitors and enhancers of iron absorption and their effect on iron status. Although the calculation of BMR was adjusted for current reported physical activity levels, the calculation was not adjusted for other factors that may affect energy metabolism, such as fat-free mass, cardiorespiratory fitness, or ethnicity. Furthermore, while CRP was used with α1GP to correct ferritin levels for inflammation [23], as CRP is an acute phase reactant, the measurement of hsCRP would have strengthened the ability of this study to assess the true level of inflammation in individuals with chronic inflammation, such as in obesity. The need to calculate the HI and NHI content of foods was primarily due to a lack of recent Australia specific food data including HI and NHI [10,25]. This was a limitation of the study as it resulted in reliance on data from 1997 [8] which fails to account for recent agricultural developments or changes to practices which may alter HI content of meats [36,60].

## 5. Conclusions

Approximately one third of the women in this study had sub-optimal iron status, confirming that ID and IDA are ongoing problems for young Australian women during reproductive years. The study found that SF levels of healthy young women were positively associated with both HI and NHI. Low total energy intake as a result of restrained eating was also found to have a negative association with iron status. Although the evidence here is associative, this may be taken to suggest that to maintain optimal SF levels, young women in Australia should be encouraged to consume iron-rich foods and to refrain from unnecessary energy restriction, or to seek professional dietetic advice when energy is restricted to assist in optimizing intake of iron and other micronutrients. As HI was a stronger predictor of SF than NHI, the study also suggests that sources of HI, particularly red meats (beef and lamb), which have a higher HI content than white meats (chicken and pork), fish, and other flesh products, may be valuable for maintaining iron status. However, further studies are needed to confirm the effect of alterations to intake of red meat on biochemically-assessed iron status. In the case of NHI and particularly for vegetarians who exclude HI sources, adequate intake of NHI, and meal planning to optimise enhancers and minimize inhibitors should be considered.

## Figures and Tables

**Table 1:** Participant characteristics of IR and ID/IDA participants.

Characteristic	Total Group (*n* = 270)	IR (*n* = 183)	ID/IDA (*n* = 87)	*p*-Value ^1^
**Age (years)**	25.9 ± 5.0	26.3 ± 4.9	25.3 ± 5.2	0.131
**Highest Educational Attainment ^2^ (*n*, %)**				
Secondary	75 (27.8)	41 (22.4)	34 (39.1)	0.014
Certificate/Diploma	43 (15.9)	33 (18.0)	10 (11.5)	
Higher education	152 (56.3)	109 (59.6)	43 (49.4)	
**Location (*n*, %)**				
Rural	89 (33.0)	66 (36.1)	23 (26.4)	0.116
Urban	181 (67.0)	117 (63.9)	64 (73.6)	
**Ethnicity (*n*, %)**				
Asia	44 (16.3)	29 (15.8)	15 (17.2)	0.362
Europe	186 (68.9)	123 (67.2)	63 (72.4)	
Other *	40 (14.8)	31 (16.9)	9 (10.3)	
**Weight Status (*n*, %)**				
Obese weight	126 (46.7)	89 (48.6)	37 (42.5)	0.347
Normal weight	144 (53.3)	94 (51.4)	50 (57.5)	
**Weight (kg)**	77.9 ± 23.8	79.0 ± 23.4	75.7 ± 24.5	0.285
**BMI (kg/m^2^)**	28.5 ± 8.6	28.8 ± 8.3	27.9 ± 9.4	0.453
**Waist circumference (cm)**	84.4 ± 18.6	85.3 ± 18.7	82.5 ± 19.1	0.252
**Activity (MET-min/week)**				
Total	2573 ± 2137	2533 ± 2079	2658 ± 2263	0.654
**Dietary Restraint (*n*, %)**				
Low	81 (30.0)	57 (31.1)	24 (27.6)	0.368
Medium	117 (43.3)	82 (44.8)	35 (40.2)	
High	72 (26.7)	44 (24.0)	28 (32.2)	
**Energy Reporting (*n*, %)**				
Low energy reporting	156 (57.8)	95 (51.9)	61 (70.1)	0.005
Plausible energy reporting	114 (42.2)	88 (48.1)	26 (29.9)	
**Oral Contraceptive Pill (*n*, %) ^**				
Yes	76 (32.3)	52 (32.7)	24 (31.6)	0.863
No	159 (67.7)	107 (67.3)	52 (68.4)	

^1^ Significance set at 5% (*p* ≤ 0.05); ^2^ Secondary education = high school completion, certificate/diploma = technical and further education or other educational institutions, higher education = tertiary or postgraduate degree; * mixed ethnicity; chi-squared tests and independent samples *t*-tests used; ^^^ incomplete sample size (*n* = 235); Abbreviations; BMI, body mass index; ID, iron deficient; IDA, iron deficient anaemia; IR, iron replete; MET, metabolic equivalent of task.

**Table 2:** Mean daily nutrient and food group intake (energy-adjusted).

Nutrient/Food Group	Total Group (*n* = 270)	IR (*n* = 183)	ID/IDA (*n* = 87)	*p*-Value ^1^
**Energy (kJ)**	7484 ± 3028	7863 ± 3145	6687 ± 2609	0.003
**Energy (kcal)**	1790 ± 724	1881 ± 752	1648 ± 624	0.003
**Nutrients**				
Protein (g)	90.9 ± 15.2	91.9 ± 15.3	89.0 ± 14.9	0.142
Fat (g)	75.5 ± 9.3	76.1 ± 9.1	74.1 ± 9.7	0.106
Carbohydrate (g)	187.7 ± 26.0	186.3 ± 27.3	190.7 ± 22.9	0.198
Fibre (g)	21.2 ± 5.5	21.2 ± 5.7	21.3 ± 5.3	0.858
Total iron (mg)	12.7 ± 2.6	12.9 ± 2.9	12.2 ± 1.9	0.012 *
HI (mg)	2.0 ± 0.9	2.1 ± 1.0	1.8 ± 0.8	0.043
NHI (mg)	10.7 ± 2.7	10.8 ± 3.0	10.3 ± 2.0	0.102 *
Zinc (mg)	11.8 ± 2.2	12.0 ± 2.4	11.3 ± 1.7	0.008 *
Calcium (mg)	882.5 ± 244.6	875.4 ± 244.4	897.4 ± 245.7	0.492
Folate (μg)	251.1 ± 63.5	255.6 ± 67.3	241.7 ± 53.7	0.092
Vitamin C (mg)	104.6 ± 49.2	105.1 ± 54.2	103.6 ± 36.7	0.813
Sodium (mg)	2404 ± 426	2424 ± 421	2363 ± 436	0.271
**Food groups (serves)**				
Fruit	1.8 ± 1.0	1.7 ± 1.0	1.8 ± 1.0	0.412
Vegetables	1.9 ± 1.0	2.0 ± 1.0	1.9 ± 0.9	0.585
Meat	1.9 ± 1.0	2.0 ± 1.1	1.7 ± 1.0	0.058
Red meat	0.7 ± 0.8	0.8 ± 0.9	0.6 ± 0.7	0.115
Poultry	0.5 ± 0.4	0.5 ± 0.4	0.5 ± 0.3	0.349 *
Fish	0.4 ± 0.4	0.4 ± 0.4	0.4 ± 0.3	0.977
Meat alternatives	0.5 ± 0.4	0.5 ± 0.4	0.5 ± 0.4	0.235
Dairy	1.7 ± 0.7	1.7 ± 0.7	1.8 ± 0.7	0.732
Grains & Cereals	3.6 ± 1.5	3.6 ± 1.7	3.6 ± 1.1	0.675 *
Discretionary	4.0 ± 1.6	4.0 ± 1.7	4.1 ± 1.3	0.647
Alcohol	0.8 ± 1.1	0.9 ± 1.2	0.6 ± 0.8	0.298 **

^1^ Significance set at 5% (*p* < 0.05); Independent samples *t*-tests were used unless denoted otherwise; * Welch’s test; ** Independent Samples Median Test. Abbreviations: ID, iron deficient; IDA, iron deficient anaemia; IR, iron replete; HI, haem iron; NHI, non-haem iron.

**Table 3:** General linear model (GLM) for SF, predictors with and without energy.

Potential Confounders	GLM (Unadjusted)			GLM (Energy Adjusted)		
	*β*	SE	*p*-Value ^1^	*β*	SE	*p*-Value ^1^
Age (years)	0.003	0.009	0.723	0.003	0.009	0.736
Education						
Secondary	−0.296	0.101	0.004	−0.292	0.102	0.004
Certificate/Diploma	0.055	0.125	0.663	0.050	0.125	0.690
Higher	0 ^a^			0 ^a^		
BMI (kg/m^2^)	0.003	0.006	0.627	0.000	0.006	0.953
Energy reporting *						
Low energy reporting	−0.181	0.090	0.044	−0.090	0.139	0.515
Plausible reporting	0 ^a^			0 ^a^		
HI **(mg)	0.125	0.049	0.010	0.128	0.049	0.009
NHI **(mg)	0.037	0.017	0.029	0.037	0.017	0.028
Energy (MJ)				0.019	0.022	0.393
Adjusted *R* Squared	0.059			0.058		

^1^ Significance set at 5% (*p* < 0.05); ^a^ Referent category; * Low energy reporting was determined using the Goldberg cut-off or 1.1 [41]; ** adjusted for energy; SE, standard error.

## Appendix A

**Table A1:** Mean daily nutrient and food group intake.

Nutrient/Food Group	Total Group (*n* = 270)	IR (*n* = 183)	ID/IDA (*n* = 87)	*p*-Value ^1^
**Energy (kJ)**	7484 ± 3028	7863 ± 3145	6687 ± 2609	0.003
**Nutrients**				
Protein (g)	91.2 ± 36.8	96.3 ± 37.7	80.4 ± 32.4	0.001
Fat (g)	75.7 ± 34.5	80.5 ± 35.2	65.6 ± 30.7	0.001
Carbohydrate (g)	187.4 ± 77.6	195.1 ± 82.3	171.2 ± 64.1	0.010 *
Fibre (g)	21.0 ± 8.5	21.8 ± 8.6	19.4 ± 8.1	0.034
Total iron (mg)	12.8 ± 5.2	13.6 ± 5.4	11.1 ± 4.3	<0.0005 *
HI (mg)	2.0 ± 1.3	2.2 ± 1.4	1.6 ± 1.1	<0.0005
NHI (mg)	10.8 ± 4.5	11.4 ± 4.7	9.5 ± 3.7	<0.0005 *
Zinc (mg)	11.7 ± 4.8	12.5 ± 5.0	10.1 ± 4.0	<0.0005 *
Calcium (mg)	882.1 ± 310.3	898.9 ± 311.2	846.8 ± 307.2	0.197
Folate (ug)	250.8 ± 90.6	263.4 ± 93.2	224.4 ± 79.2	0.001
Vitamin C (mg)	104.3 ± 54.7	107.8 ± 59.6	97.0 ± 42.2	0.130
Sodium (mg)	2404 ± 1071	2547 ± 1083	2104 ± 987	0.001
**Food groups (serves)**				
Fruit	1.6 ± 1.1	1.6 ± 1.1	1.6 ± 1.0	0.941
Vegetables	2.0 ± 1.0	2.0 ± 1.0	1.9 ± 0.9	0.364
Meat	2.9 ± 1.4	2.1 ± 1.4	1.5 ± 1.1	<0.0005 *
Red meat	1.0 ± 0.9	1.2 ± 1.0	0.8 ± 0.7	<0.0005 *
Poultry	0.5 ± 0.5	0.6 ± 0.5	0.4 ± 0.3	0.098 **
Fish	0.4 ± 0.4	0.4 ± 0.4	0.3 ± 0.3	0.662 **
Meat alternatives	0.5 ± 0.4	0.5 ± 0.4	0.5 ± 0.4	0.945
Dairy	1.7 ± 0.8	1.8 ± 0.8	1.7 ± 0.8	0.619
Grains and Cereals	4.0 ± 2.3	4.2 ± 2.5	3.5 ± 1.7	0.008
Discretionary	3.7 ± 2.5	3.9 ± 2.6	3.3 ± 2.2	0.062
Alcohol	0.8 ± 1.1	0.9 ± 1.2	0.6 ± 0.8	0.055 **

^1^ Significance set at 5% (*p* < 0.05), IR vs. ID/IDA; Independent samples *t*-tests used unless otherwise denoted; * Welch’s Test; ** Independent Samples Median Test; Participants were categorised as being iron replete (IR) (SF > 20 µg/L and Hb > 120 g/L, *n* = 183) or iron deficient/iron deficient anaemic (ID/IDA) (ID = SF < 20 µg/L, IDA = SF < 20 µg/L and Hb < 120 g/L, *n* = 87).
